# The rare Hematological disorder; A man with Hemophagocytic Lymphohistiocytosis (HLH)

**DOI:** 10.22088/cjim.12.0.439

**Published:** 2021

**Authors:** Majid Gholizadeh, Shirin Kianersi, Leila Noorazar, Vahid Kaveh, Elham Roshandel, Sina Salari

**Affiliations:** 1 Hematopoietic Stem Cell Research Center, Shahid Beheshti University of Medical Sciences, Tehran, Iran; 2 Department of Hematology and Blood Banking, School of Allied Medical Sciences, Shahid Beheshti University of Medical Sciences, Tehran, Iran; 3 department of adult Hematology & Oncology ,School of Medicine, Ayatollah Taleghani Hospital, Shahid Beheshti University of Medical Sciences, Tehran, Iran; 4 Firouzgar Hospital, Iran University of Medical Sciences, Tehran, Iran; 5 Department of Hematology and Medical Oncology, Faculty of Medicine, Iran University of Medical Sciences, Tehran, Iran

**Keywords:** Hemophagocytic Lymphohistiocytosis, Splenomegaly, Fever

## Abstract

**Background:**

Hemophagocytic lymphohistiocytosis (HLH) is a rare disease with different causes. HLH has been categorized into two sub-groups; primary HLH which is associated with some gene mutations and secondary HLH that is developed by various causes, such as autoimmune disease, infections, and malignancies. However, the symptoms of both groups are identical and if left untreated, it will result in death.

**Case Presentation:**

In this study, we reported a 39 years old man had symptoms such as fever, weakness and chill for a month period of time. Firstly, due to pancytopenia in peripheral blood findings and clinical manifestations, he had been diagnosed with myelodysplastic syndrome (MDS) with an excess blast but the elevated liver enzymes and bilirubin were not consistent with this diagnosis. Hence, we recommended more investigation such as CT scan, bone marrow aspiration and bone marrow biopsy with immunohistochemistry tests. Finally, we found macrophages and histiocyte in bone marrow biopsy smear with Wright-Giemsa staining that engulfed the cells such as platelets and lymphocytes, so HLH syndrome was confirmed and treatment program with latest approved protocols started for the patient.

**Conclusion:**

HLH syndrome is a life-threatening disease that can be saved if timely diagnosed. Therefore, more consideration of all the laboratory findings and clinical signs of the patient can help to diagnose the disease more accurately. Also, we did a review of its pathophysiology, symptoms and therapeutic treatments.

Hemophagocytic lymphohistiocytosis (HLH) is a rare, life-threatening syndrome characterized by severe immune activation and dysregulation (1). The pathophysiology of HLH is based on the deficiency of natural killer (NK) cells and cytotoxic T lymphocytes (CTLs) in their cytotoxic functions (2). HLH can be developed at any age and classified into primary and secondary. Primary HLH is inherited and symptoms appear during infancy. The secondary HLH is related to a variety of underlying diseases that affect normal immune responses such as malignancy, hence its occurrence in healthy individuals is scarce (3). Clinical manifestations of HLH include high fever, pancytopenia, liver dysfunction, and the pathological finding of hemophagocytosis in the bone marrow and other tissues. HLH diagnosis is a challenge because signs are often nonspecific and must be distinguished from severe sepsis (4). 

The study was approved by the ethical committee of Shahid Beheshti University of Medical Sciences, Tehran, Iran (IR.SBMU.RETECH.REC.1399.607). 

## Case presentation

A 39-year-old man with no history of disease was referred to the physician for fever, chills, and weakness. The patient said to have had no contact with ticks or insects but has had close contact with domestic livestock in recent days. In physical examinations, the temperature was 40°C and there was splenomegaly. Laboratory tests showed that the patient had pancytopenia with white blood cells (WBCs): 900 cells/µL (normal range: 4-1110^3 ^cells/µL), red blood cells (RBCs): 3.7310^6^cells/µL (normal range: 4.5-5.510^6 ^cells/µL), platelet (Plt): 72000/µL, (normal range: 150-40010^3^/µL). Furthermore, the initial investigations at the time of presentation are shown in table 1. Diagnostic tests for infections were reported as follows; HBS Ag: nonreactive, HBS antibody (HBS Ab): 1 IU/L, HBC Ab: nonreactive, HCV: nonreactive, HIV: nonreactive, HAV: 1.35 IU/L, HBC Ab: nonreactive. Ultrasound scan of the abdomen and pelvis revealed huge splenomegaly (193*88 mm) with normal parenchyma. Doppler Ultrasound showed that the hepatic artery was enlarged and had a resistive index (RI)=0.8. Portohepatic lymphadenopathy and para-aortic lymphadenopathy with size up to 3020 mm were seen. On physical examination, no palpable lymphadenopathy was found. Other organs including the liver, bladder, gallbladder, bile ducts and pancreas were normal in ultrasound imaging. Moreover, in cardiovascular consultation, endocarditis was rejected. 

During the hospitalization, blood cultures were negative for two times, indicating no blood infection in the patient. All of the diagnostic tests for brucellosis, including Wright, coombs-wright test and 2-ME test were negative. Direct and indirect Coombs tests for hemolytic anemia diagnosis were also negative. Also, reticulocyte count and reticulocyte production index (RPI), respectively were 0.8% and 0.22; therefore, the possibility of hemolytic anemia was ruled out. The systemic lupus erythematosus (SLE), rheumatoid arthritis (RA) and other autoimmune diseases screening tests were performed by the following immunologic panel (table 1) and eventually, the possibility of autoimmune diseases was ruled out. According to the symptoms of splenomegaly and lymphadenopathy, a hematologic neoplasm was suspected. Bone marrow aspiration (BMA) and bone marrow biopsy (BMB) were performed to find the signs of leukemia. Microscopic examination of peripheral blood smear showed hypochromic, anisocytosis and severe leukopenia with mild lymphocytosis without any blasts. 

**Table 1:** Initial investigations at the time of presentation

Initial investigation	Value	Reference range
**WBC**	900/µL	4000-10000/µL
**RBC**	3.7310^6^/µL	3.9-5.810^6^/µL
**PLT**	72000/µL	150000-450000/µL
**Hemoglobin**	8.9 g/dL	11-17 g/dL
**Hematocrit**	30%	33-53%
**ESR**	64 mm/h	0-22 mm/h
**CRP**	2+	Negative
**AST**	324 IU/L	0-37 IU/L
**ALT**	202 IU/L	0-41 IU/L
**ALP**	914 U/L	80-306 U/L
**LDH**	1058 IU/L	230-450 IU/L
**BUN**	17 mg/dL	7-20 mg/dL
**Creatinine**	1.3 mg/dL	0.7-1.4 mg/dL
**Total Bilirubin**	6.9 mg/dL	0.1-1.2 mg/dL
**Direct Bilirubin**	5 mg/dL	Up to 0.4 mg/dL
**Triglyceride**	340 mg/dL	40-160 mg/dL
**Ferritin**	>1500ng/mL	27-375 ng/mL
**C3**	84 mg/dL	90-180 mg/dL
**C4**	13 mg/dL	10-40 mg/dL
**IgG**	971 mg/dL	Adult: 700-1600 mg/dL
**RF**	Negative	
**HBC IgM**	0.1 mg/dL	<5 Negative
**CH50**	80	Absence/low:0-50 Normal:51-150 High:>151
**ANA**	0.3 IU	Negative:<0.9 IU Borderline: 0.9-1.1 IU Positive: >1.1 IU
**Anti-dsDNA**	2.5 U/mL	Negative: <16 Borderline: 16-24 Positive: >24
**Anti-CCP**	4.7 U/mL	Normal: <16 Equivocal:16-24 Positive: >24
**Pr-3**	0.6 U/mL	Negative: <12 Equivalent: 12-18 Positive: >18
**MPO**	1.9 IU/mL	Negative: <3.5 IU/mL Equivalent: 3.5-5 IU/mL Positive: >5 IU/mL
**Direct coombs**	Negative	
**Indirect coombs**	Negative	

WBC. White blood cell; RBC. Red blood cell; PLT. Platelet; ESR. Erythroid sedimentation ratio; CRP. C-reactive protein; AST. Aspartate aminotransferase; ALT. Alanine aminotransferase; ALP. Alkaline phosphatase; LDH. Lactate dehydrogenase; BUN. Blood urea nitrogen; C3, C4. Complement system proteins; IgG. Immunoglobulin G; RF. Rheumatoid factor; HBC IgM. Immunoglobulin M against Hepatitis B core antigens; CH50. 50% of the hemolytic function of the complement system; ANA. Anti-nuclear antibody; Anti-dsDNA. Antibody against double-strand DNA; Anti-CCP. Anti-cyclic citrullinated peptide; Pr-3. Proteinase 3; MPO. Myeloperoxidase BM examination showed erythroid, myeloid and megaloid cells, dysplastic changes, maturation arrest and myeloid/erythroid (M/E) ratio: 1/3 (severe erythroid hyperplasia). Eighteen to twenty percent of observed lymphocyte cells were small. Also, a few smudge cells with a large number of bare nuclei, 5-6% blasts and megakaryocytes with dysplastic appearance were observed. Bone marrow biopsy revealed that the bone trabeculae are occupied (80%) by erythroid precursors and megakaryocytes had a dysplastic appearance. Immunohistochemistry (IHC) findings showed that CD117^+^ in <10% blast, CD34^+^ in rare blasts, CD3 was positive in few scattered lymphocytes and lymphoid aggregates, and myeloid series were positive for MPO. Therefore, MDS with excess blasts was diagnosed at first but splenomegaly and elevated liver enzymes remained unjustified. BM film clearly showed hemophagocytic appearance in which erythrocytes, leukocytes, platelets, and their precursors were phagocytized by histiocytes and crowded macrophages.

**Table 2:** HLH diagnosis criteria based on the Histiocyte Society in 2004

Criteria	Present in this patient
**Fever(temperature >38.5 ºC for >7days**	Yes
**Splenomegaly**	Yes
**Cytopenia involving 2 or more lines **	Yes
**Hypertriglyceridemia and/or hypofibrinogenemia ** **(fasting triglyceride levels >3 mmol/L** **fibrinogen <1.5 g/L**	Yes (Hypertriglyceridemia)
**Ferritin level >500 µg/L**	Yes
**Soluble CD25 level >2400 U/mL**	Not measured
**Decreased or absent natural killer cell activity**	Not measured
**Hemophagocytosis in bone marrow, central nervous system, or lymph nodes**	Yes

## Discussion

HLH can be classified as primary and secondary. Secondary HLH (acquired HLH) more commonly appears in adults and is associated with other acquired underlying conditions (5). Studies on the secondary HLH showed that infection was the most common underlying etiology (41.1%), followed by malignancy (28.8%), autoimmune diseases (6.8%) and post solid organ transplantation (2.7%). However, unknown causes comprise a large subset (17.8%) of the documented cases (6). HLH can be idiopathic without a known underlying condition, but it is critical to differentiate between primary and secondary HLH to choose suitable treatment. The mortality rate of untreated HLH is high with a median survival of fewer than 2 months (7).

**Diagnosis: **The diagnosis of HLH is difficult because the clinical features can be non-specific. Therefore, misdiagnosis is common. According to HLH diagnosis criteria determined by the Histiocyte Society in 2004 (8), which is shown in table 2, at least five of the eight criteria must be present for a diagnosis to be established. Our case fulfilled six features out of eight diagnostic criteria for HLH diagnosis. According to the study from China on 103 patients with HLH syndrome, the prevalence of clinical signs of HLH syndrome was determined as follows: >96% of patients with HLH presented with high-grade fever, while 79.6% had splenomegaly and 53.4% had lymphadenopathy. Among the laboratory findings, 98.4% of the patients had a significant elevation of serum ferritin (≥500 μg/L). Cytopenia in 98% of the patients was found, bone marrow hemophagocytosis was seen in 87.4% of patients and hypertriglyceridemia was noted in 85% of the patients (9).

Visceral Leishmaniasis is one of the endemic parasitic infections in Iran that can has clinical symptoms like HLH syndrome (10). In our case, the absence of Leishman's body in the bone marrow biopsy slide, the negative serological results as well as no history of any insects’ bites, ruled out leishmaniasis. Our case was suspected for MDS at first because the BM examination showed erythroid and myeloid with megaloid and dysplastic changes. Maturation arrest (5-6% blasts) and megakaryocytes with dysplastic appearance were observed. Erythroid precursors had occupied (80%) the bone trabeculae and IHC tests all reported in favor of MDS diagnosis, but the clinical and laboratory features such as splenomegaly and elevated liver enzymes contradicted with the MDS, so it was excluded. Later with more investigation at BM smear, we rarely found some macrophages that phagocytosed mononuclear cells, including lymphocytes, platelets, and erythroid precursors (Fig. 1). Based on the previously mentioned criteria and BM smear, the HLH was confirmed.

**Etiology: **Among viral infections were the most frequent infections to cause secondary HLH disease. Our patient was negative for EBV, HIV, hepatitis B, C, and cytomegalovirus (CMV). Autoimmune diseases such as systemic lupus erythematosus (SLE) and rheumatoid arthritis (RA) were ruled out according to the immunological panel mentioned in table 1. Also, we suspected hemolytic autoimmune disease due to daily drop-in blood cells count along with an increase in blood bilirubin, but the negative direct and indirect Coomb’s test and elevated liver enzymes did not confirm it.

Malignancy is one of the underlying causes that may lead to HLH syndrome. In this case, CT scan showed unpalpable bilateral axillary lymphadenopathy, thus we suspected lymphoma. Due to the limitation of access to the mesenteric lymph nodes, we could not perform biopsy and diagnostic tests. Hence, malignancy was neither confirmed nor ruled out. Therefore, in this case, HLH was diagnosed but the underlying cause of HLH remained unknown. 

**Treatment: **According to the treatment protocols for HLH (8), the patient was started on etoposide (150 mg/m^2 ^of body area) along with dexamethasone (20 mg/day) for 8 weeks. Because of the liver's inability in drug detoxification and hyperbilirubinemia, the initial dose of etoposide was 200 mg/m^2^ and continued with 150 mg/m^2^. The investigation of changes in the patient’s laboratory parameters during his admission is shown in Fig.1.

**In conclusion **HLH syndrome is a life-threatening disease, if not correctly diagnosed, it will surely cause death. So it is critical to diagnose the disease at the early stage to save the patient’s life. 
